# Palliative care pathways in Amyotrophic Lateral Sclerosis (ALS): a sequence analysis of health claims data

**DOI:** 10.1186/s12904-025-01843-x

**Published:** 2025-07-14

**Authors:** Richard Schmidt, Ekaterina Slotina, Franziska Meissner, Moritz Metelmann, Benjamin Ilse, Verena Vogt, Antje Freytag

**Affiliations:** 1 University Hospital Jena, Institute of General Practice and Family Medicine, Friedrich Schiller University Jena, Jena, Germany; 2 Leipzig University, Faculty of Medicine, Department of Neurology, Leipzig, Germany; 3 University Hospital Jena, Department of Neurology, Friedrich Schiller University Jena, Jena, Germany

**Keywords:** Palliative care, Palliative medicine, Amyotrophic lateral sclerosis, Secondary data analysis, Hospice care

## Abstract

**Background:**

Amyotrophic lateral sclerosis is a progressive neurodegenerative disease requiring palliative care. Despite the availability of palliative care services, utilization patterns among people with ALS (pALS) remain poorly understood. This study aimed to analyze palliative care utilization (i.e., primary palliative care (PPC), specialized palliative homecare (SPHC), inpatient palliative care, hospice services) in the last year of life among pALS, to identify distinct care pathways using sequence analysis, and examine their association with end-of-life care quality.

**Methods:**

A retrospective cross-sectional study using German health claims data (2016 – 2019). Sequence analysis with Temporal Needleman-Wunsch alignment and clustering identified pathway clusters based on type, order, and timing of palliative care services. The study included 1,295 pALS who died between 2016 and 2019 and were insured with a large German health insurance provider. Inclusion required an ALS diagnosis without concurrent cancer.

**Results:**

Of 1,295 pALS, 695 (53.7%) received palliative care. Sequence analysis identified nine distinct care pathway clusters. Quality indicators varied highly across clusters. Pathways involving SPHC, either alone, with PPC, and/or with hospice care, showed fewer emergency visits, hospital stays, and in-hospital deaths, suggesting higher end-of-life care quality.

**Conclusions:**

Palliative care utilization varies substantially in type, order, and timing. Findings suggest that end-of-life care quality depends not only on the provision of palliative care but also on when and on how different services are combined. Future research should examine the role of interdisciplinary collaboration in palliative care pathways and explore preferences and clinical characteristics of pALS to better understand factors influencing end-of-life care quality.

## Background

Amyotrophic lateral sclerosis (ALS) is a progressive neurodegenerative disorder characterized by the degeneration of motor neurons, leading to severe physical disability. Due to its rapidly fatal course, with a median survival time of 2 to 4 years, ALS imposes complex psychosocial and medical challenges on people with ALS (pALS), their informal care givers, and health care professionals [1–3]. Complementing well established palliative care concepts and guidelines of other disciplines such as oncology, emerging concepts in neuropalliative care target the distinct challenges imposed by neurologic diseases like ALS [4, 5]. For example, ALS’ rapid disease progression demands timely and informed decision-making, while the variability in symptoms requires highly individualized approaches [6]. Additionally, healthcare professionals often perceive the disease’s terminal phase, commonly marked by respiratory paralysis, as different from other fatal diseases, complicating its recognition and management [7]. Nevertheless, neuropalliative care shares the fundamental goals of palliative care in other disciplines, namely improving quality of end-of-life care, for example by reducing burdensome treatment and supporting terminally ill to die in their preferred environment, typically outside of institutional settings such as hospitals.

In countries with universal health coverage and well-developed healthcare infrastructures, a range of services targets the needs of people with terminal diseases like ALS. In Germany, these services are provided either at patients’ residences (including hospices and nursing homes) or in hospitals. Primary palliative care (PPC) is provided predominantly by general practitioners with or without additional certification in palliative medicine collaborating with nursing or hospice teams. Specialized palliative home care (SPHC), delivered by a multidisciplinary team of specialized physicians and nurses with expertise in palliative care, may be offered independently or in addition to PPC [8–10]. Furthermore, inpatient palliative care units and hospices offer comprehensive institutionalized palliative care, particularly during advanced or terminal stages of disease. Despite the availability of these four distinct services (i.e., PPC, SPHC, inpatient palliative care unit, hospice) palliative care appears to remain underutilized among pALS [11] and the pathways of those with neurological disease navigating across palliative care structures remain poorly understood [12, 13].

This study aimed to address these gaps by analyzing routinely collected German health claims data from BARMER [14], one of the largest statutory health insurance funds in Germany covering more than 10% of the German population, to explore palliative care utilization during the last year of life among pALS. We employed novel analytical approaches to analyze the palliative care path of pALS, that is the longitudinal pathway in regards to palliative care services provided to individuals [15]. Specifically, first we described types and proportions of palliative care services utilized. Secondly, we hypothesized that palliative care pathways can be clustered based on the type, order, and timing of palliative care initiation. Therefore, we operationalized pathways as linear sequences and applied sequence analysis to align and cluster them into distinct groups. Finally, we investigated how quality of end-of-life care varied across clustered palliative care sequences.

## Methods

### Study design

In this retrospective cross-sectional study, we utilized administrative health claims data of adults insured with BARMER health insurance, who died within the four-year-period from 2016 to 2019. BARMER health insurance is a major German health insurance provider, covering more than 10 percent of all people with statutory health insurance in Germany.

This study was part of the research project *pallCompare*, registered in the German Clinical Trial Register on 28 June 2021 (ref.-no. DRKS00024133). It was conducted in accordance with the guidelines of the Declaration of Helsinki and was approved by the ethics committee of Jena University Hospital (reg.-no. 2021–2162-Daten). The study followed RECORD Reporting Guidelines for studies using routinely-collected health data [16].

### Data

The administrative claims data provided by BARMER as part of a broader research project *pallCompare*included information on health claims coded within 365 days before any insured individual’s death. To filter for pALS we selected all individuals with ICD-10 G12.2 coded at least once (confirmed outpatient or hospital diagnosis) within the last year of life. Subsequently, we removed cases with a concordant outpatient or inpatient diagnosis of cancer (ICD-10 C00.* – C97.*) to exclude a major competing source for palliative care demand [17].

For all included pALS, we analyzed sociodemographic and clinical variables including age, sex, and the weighted Charlson Comorbidity Index (CCI) [18] as a measure of comorbidity burden. Further, several dichotomous markers indicated whether an individual had been diagnosed with either of the following diseases: cardiovascular disease, cerebrovascular disease, kidney disease, liver disease, respiratory disease, dementia [17].

For each of the four types of palliative care (PPC, SPHC, inpatient palliative care, hospice) a continuous variable reflecting days before death (0 – 365) indicated the timepoint before death when the respective type of service had first been billed in an in- or outpatient setting, i.e., the day this type of service had been initiated.

Additionally, we recorded the place of death for each individual using four separate dichotomous variables, each corresponding to a specific location (hospital, inpatient palliative care unit, nursing home, or hospice). Each variable was coded as 1 if the individual died in the respective location and 0 otherwise. Five dichotomous variables encoded whether each of the following had been billed within 30 days before death: hospital stay, intensive care unit (ICU) stay, emergency service deployment, percutaneous endoscopic gastrostomy, and mechanical ventilation [8, 19].

The quality of end-of-life care was operationalized using indicators derived from previous research utilizing claims data [9, 20–24]. These included hospital stays, ICU stays, emergency service deployments as well as in-hospital deaths among others. While not direct measures of symptom control or patient preferences, utilization of intensive care is unlikely to align with patient preferences in palliative settings. Place of death, in particular, is a frequently used endpoint in palliative care services research and has been associated with perceived quality in both patients and caregivers [25, 26].

### Analyses

To characterize the study cohort we used descriptive statistics, including median and interquartile range for continuous variables, as well as counts and percentages for categorical data.

In this study, we analyzed palliative care pathways of pALS who had been provided with at least one type of palliative care. For each individual, we constructed linear sequences representing the order and type of up to four palliative care services: PPC, SPHC, inpatient palliative care, and hospice care. Timing indices between sequential events indicated the number of days between the initiations of subsequent services, with a value of 0 assigned if multiple services were initiated simultaneously. If a palliative care service had not been utilized, it was excluded from the individual’s sequence. We then clustered individual palliative care sequences applying a combination of sequence alignment und subsequent clustering [27, 28].

Sequence alignment is a technique traditionally used to compare biological sequences of nucleic acids. The Needleman-Wunsch (NW) algorithm is a widely used technique for global sequence alignment, designed to find the best possible alignment of two sequences across their entire lengths [29]. It maximizes the similarity score of two linear sequences by considering matches, mismatches, and gaps between elements of two sequences, e.g., letters or symbols [30]. The algorithm’s applications extend to diverse fields, such as bioinformatics and time-series data analysis, where maintaining the sequence order is crucial.

The Temporal Needleman-Wunsch (TNW) algorithm adapts the NW framework for longitudinal data by introducing temporal dynamics into the alignment process [31]. TNW incorporates the transition time information between sequence elements and penalizes discrepancies in the timing of elements. This makes TNW valuable for longitudinal healthcare datasets, where not only the order but also the timing of elements representing medical events, e.g., interventions, holds significant importance.

Incorporating the capabilities of TNW, the software package AliClu facilitates clustering sequences to identify subgroups of individuals with similar longitudinal data [32]. Using TNW for sequence alignment, AliClu computes a similarity matrix representing pairwise similarity scores for all input sequences. This similarity matrix is then transformed into a dissimilarity or distance matrix, which serves as the basis for agglomerative hierarchal clustering. This clustering approach does not require a predefined number of clusters [33]. Instead, AliClu evaluates a specified range of possible cluster numbers and determines the optimal solution based on clustering indices and cluster stability measures, which are detailed in Rama et al. [32]. Thus, by entering the sequences and a range of potential numbers, AliClu automatically identifies the optimal number of clusters within a prespecified range and assigns sequences accordingly. In our study, sequences varied in length and could range from a single palliative care service to multiple events involving different types of palliative care. They were aligned and clustered using AliClu.

We used descriptive statistics, including median and interquartile range for continuous variables, as well as counts and percentages for categorical data to summarize and compare data across clusters. All statistical analyses on our cohort’s characteristics were performed using R Statistical Software (Version 4.4.2) with R Studio (Version 2024.09.1 + 394) [34, 35].

## Results

The study cohort included 1,295 pALS without any concurrent diagnosis of cancer (79.59% of all pALS), who died within the 4-year-period from 2016 to 2019. As shown in Table 1, palliative care had been provided to 695 pALS (53.7%). Among those with palliative care, there was a higher proportion of females (416, 59.9%) compared to males, while almost no difference in sex proportions was seen among those without palliative care (311 females, 51.8%). The median age was similar among those with and without palliative care (72 years and 74 years respectively). pALS without palliative care exhibited a higher CCI and a greater prevalence of comorbidities compared to those with palliative care and the overall study cohort.

**Table 1:** Demographic and clinical characteristics of the study cohort by palliative care utilization

Characteristic [metrics]	Full population	With palliative care	Without palliative care
	(*N* = 1295)	(*N* = 695)	(*N* = 600)
Sex, female [n (%)]	727 (56.1)	416 (59.9)	311 (51.8)
Age, years [median (IQR)]	73 (65 – 79)	72 (64 – 78)	74 (66 – 80)
Charleston Comorbidity Index, weighted [median (IQR)]	3 (2 – 5)	3 (2 – 5)	4 (2 – 6)
Diagnoses^a^ [n (%)]			
Cardiovascular disease	1096 (84.6)	568 (81.7)	528 (88.0)
Respiratory disease	877 (67.7)	457 (65.8)	420 (70.0)
Cerebrovascular disease	419 (32.4)	192 (27.6)	227 (37.8)
Dementia	389 (30.0)	174 (25.0)	215 (35.8)
Kidney disease	317 (24.5)	141 (20.3)	176 (29.3)
Liver disease	175 (13.5)	88 (12.7)	87 (14.5)

^a^ diagnoses defined according to Murtagh et al. *Palliat Med.* 2014

*Abbreviations*: *IQR* interquartile range

**Fig. 1:**
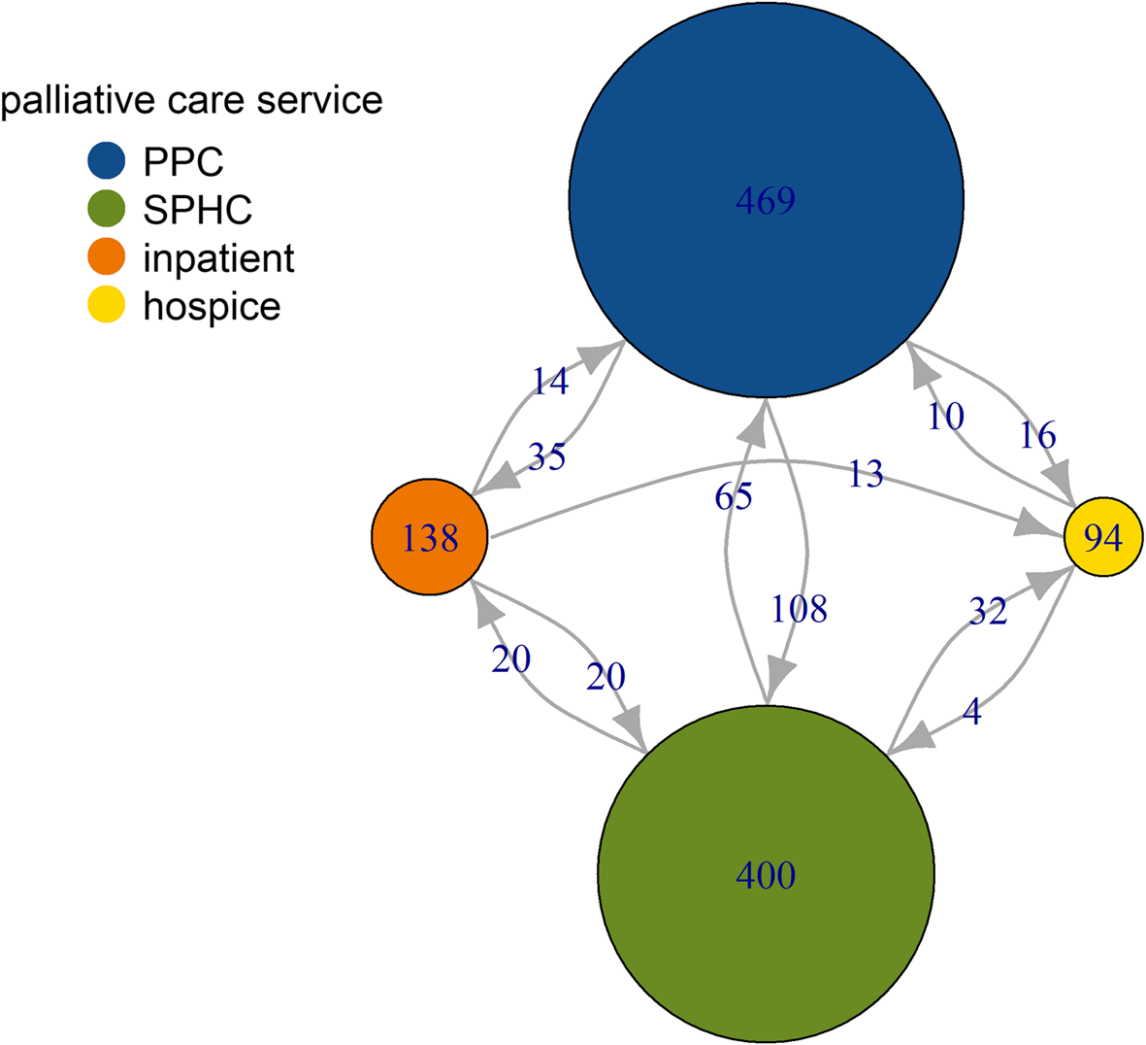
Sequential utilization of palliative care services among people with ALS in the last year of life. Vertices represent the total number of pALS who initiated the respective type of palliative care service within the last year of life. Directed edges indicate the total number of pALS who initiated the connected types of palliative care services sequentially, following the direction of the edge. Those who initiated multiple types of palliative care services simultaneously were included in the vertex labels but not represented by directed edges: 27 pALS initiated PPC and SPHC simultaneously, 12 initiated hospice care simultaneously with PPC, and 10 initiated hospice care simultaneously with SPHC. Abbreviations: PPC, primary palliative care; SPHC, specialized palliative home care; inpatient, inpatient palliative care

The majority of pALS with palliative care utilized outpatient services. As illustrated in Fig. 1, PPC was delivered to 496 pALS (71.4%), while SPHC was provided to 400 pALS (57.6%). Inpatient palliative care at a hospital had been provided to 138 pALS (19.9%), and hospice care had been delivered to 94 pALS (13.5%). Table 2 summarizes the proportions of utilization for each type of care.

**Table 2:** Summary of quality indicators and place of death across nine identified palliative care clusters in people with ALS

**Cluster** (% of all pALS with PC)	**PPC** (median^a^)	**SPHC** (median^a^)	**IPC** (median^a^)	**Hospice** (median^a^)	**30 days before death**					**Place of death**				
					**Hospital stay**	**ICU stay**	**Emergency service deployment**	**PEG**	**Mechanical ventilation**	**Hospital**	**IPC**	**Nursing home**	**Hospice**	**Other**
**1** (28.2)	100.0 (126.5)	0.0	0.0	0.0	41.8	9.7	38.3	4.1	6.6	32.1	1.0	24.5	0.0	43.4
**2** (20.4)	0.0	100.0 (31.0)	0.0	0.0	28.9	1.4	28.9	4.2	1.4	9.9	0.7	18.3	0.0	71.8
**3** (11.9)	100.0 (156.0)	100.0 (31.0)	0.0	0.0	22.9	6.0	25.3	3.6	2.4	8.4	1.2	15.7	0.0	75.9
**4** (10.6)	100.0 (60.5)	100.0 (96.5)	0.0	2.7 (365.0)	31.1	8.1	27.0	6.8	8.1	12.2	0.0	18.9	1.4	67.5
**5** (7.2)	66.0 (172.0)	74.0 (145.0)	20.0 (47.0)	100.0 (20.0)	20.0	2.0	16.0	2.0	4.0	0.0	0.0	8.0	90.0	2.0
**6** (6.3)	0.0	0.0	100.0 (5.5)	0.0	95.5	11.4	40.9	15.9	6.8	93.2	45.5	0.0	0.0	6.8
**7** (5.8)	85.0 (107.5)	52.5 (67.0)	100.0 (6.5)	0.0	87.5	2.5	40.0	10.0	5.0	77.5	30.0	2.5	0.0	20.0
**8** (5.5)	86.8 (31.0)	39.5 (166.0)	63.2 (98.5)	76.3 (28.0)	18.4	5.3	15.8	0.0	2.6	10.5	2.6	23.7	57.9	7.9
**9** (4.0)	57.1 (83.5)	100.0 (26.0)	71.4 (70.0)	46.4 (26.0)	17.9	0.0	3.6	0.0	3.6	0.0	0.0	14.3	42.9	42.8
**with palliative care (*n* = 695)**					38.0	5.9	29.6	4.9	4.6	24.3	5.3	0.0	11.5	64.2
**without palliative care (*n* = 600)**					60.3	19.7	52.0	6.3	19.8	53.5	0.7	14.5	0.0	32.0

Columns: (1) cluster number and size (% of all pALS with palliative care); (2–5) within-cluster proportions (%) and median time from initiation of palliative care type until death (^a^); (6–10) indicators for quality of end-of-life care (low rates resemble high quality); (11–15) place of death. Last two rows summarize proportions (%) of quality indicators of end-of-life care and place of death across entire groups of pALS with/without palliative care during last year of life

*Abbreviations*: *PC* palliative care, *PPC* primary palliative care, *SPHC* specialized palliative home care, *IPC* inpatient palliative care, *ICU* intensive care unit, *PEG* percutaneous endoscopic gastrostomy

### Clustering of palliative care pathways

**Fig. 2:**
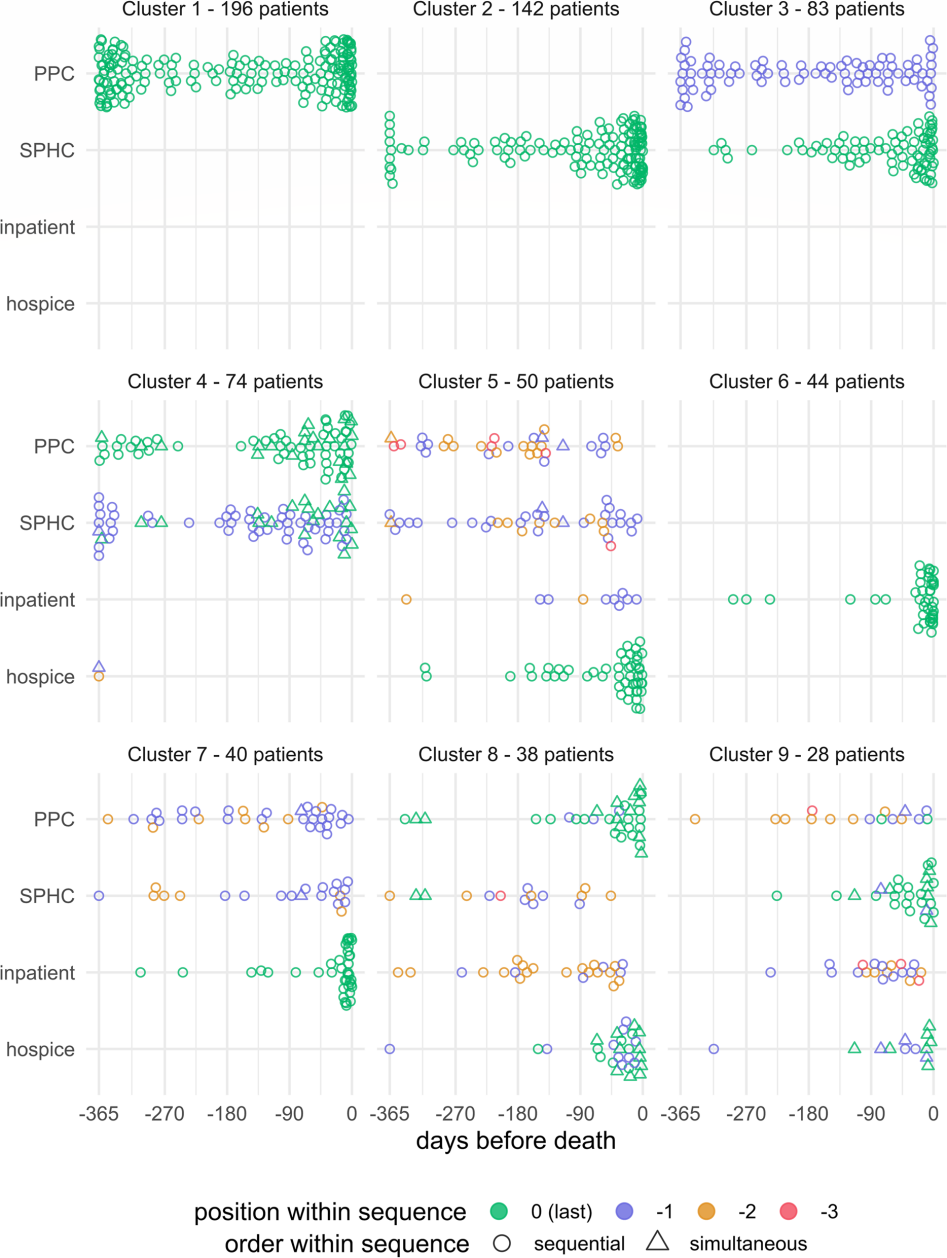
Temporal patterns of palliative care initiation in people with ALS across nine distinct clusters. Symbols within each cluster indicate the initiation of a specific type of palliative care service at the corresponding time point on the x-axis. Within an individual’s palliative care sequence, the color of the symbols reflects the order of care initiation: green (0) represents the most recent type of palliative care received, purple (−1) indicates the second-to-last type, and so on. If multiple types of palliative care services had been initiated on the same day, a triangle in both types marks the respective day. Abbreviations: PPC, primary palliative care; SPHC, specialized palliative home care; inpatient, inpatient palliative care

Using sequence analysis, the palliative care pathways of 695 pALS with palliative care were clustered into nine distinct groups (Fig. 2). More than two-thirds of pALS with palliative care fell into clusters 1 to 4, representing those with outpatient care exclusively, through either PPC, SPHC, or the sequential or simultaneous initiation of both. pALS with hospice care as their final type of palliative care, following prior initiation of PPC or SPHC, and a few pALS with inpatient palliative care before, characterized cluster 5. Importantly, 10% of pALS in cluster 5 had received hospice care as their final type of palliative care but did not decease within a hospice. Clusters 6 and 7 comprised pALS whose final type of care was inpatient palliative care. While pALS of cluster 6 had been provided with only inpatient palliative care during their last year of life, this type of palliative care was preceded by PPC or SPHC in cluster 7. Clusters 8 and 9 represented the smallest groups of pALS. Cluster 8 comprised pALS who primarily received PPC and/or hospice care as their final palliative service, often following PPC or inpatient palliative care. In contrast, nearly all pALS in Cluster 9 received SPHC as their final service, sometimes alongside hospice care and often preceded by PPC or inpatient palliative care.

Table 2 provides a quantitative overview on all clusters. Median time from the initiation of different types of palliative care to death varied across clusters. Across clusters, the median time of initiation ranged from 26 to 172 days for outpatient services, 6 to 99 days for inpatient palliative care, and 20 to 28 days before death for hospice care.

### Quality of end-of-life care

Indicators for quality of end-of-life care varied highly across clusters. Among the largest group of those only utilizing PPC and/or SPHC, clusters 2 and 3 exhibited best outcomes as shown by quality indicators in Table 2. Both incorporated SPHC at last (median time of initiation 31 days before death). Clusters characterized by late initiation of inpatient palliative care (e.g., Cluster 6) on the other hand, demonstrated the highest rate of hospital stays (95.5%), emergency service deployments (40.9%), ICU admissions (11.4%), and death in hospital (93.2%). In contrast, clusters with an emphasis on utilization of hospice care (i.e., Clusters 5, 8, 9) showed low utilization of potentially aggressive end-of-life care and low rates of deaths in hospital.

Aggregated results of pALS with palliative care revealed lower rates in all indicators of quality of end-of-life care compared than without palliative care.

## Discussion

In the period from 2016 to 2019, only 53.7% of all pALS had been provided with some type of palliative care within their last year of life, indicating potential gaps in access or awareness of palliative care services. Among pALS with palliative care, sequence analysis identified nine distinct groups of palliative care pathways, revealing substantial differences in care delivery within the overall group of pALS with palliative care. Among these, about two-thirds utilized outpatient palliative care services, either PPC, SPHC, or a combination of both initiated sequentially or simultaneously. Clusters involving SPHC, either alone, with PPC, or with hospice care, demonstrated high quality of end-of-life care, whereas those with late hospital-centered inpatient palliative care showed higher rates of emergency service deployments, hospital stays and deaths in hospital. These findings suggest that quality of end-of-life care is not only associated with whether any palliative care was provided but also differs with regard to the sequence of palliative care services.

Palliative care is increasingly recognized as a vital component of multidisciplinary management of ALS [5, 12]. While palliative care has been extensively researched and implemented in oncologic conditions, its integration in non-malignant diseases remains limited and underresearched [36–38]. Narrowing this research gap, our study provided insights into palliative care of pALS. Making use of routinely collected administrative health claims we were able to cluster palliative care pathways and assess their variability with regards to quality of end-of-life care.

Our results suggest that – similar to other non-malignant diseases – the integration of palliative care corresponds with increased end-of-life care quality in pALS [39, 40]. At the same time the observed underutilization of palliative care in this rapidly fatal disease might indicate poor awareness and accessibility of palliative care services among pALS, informal caregivers, and health care professionals. Importantly, pALS who did not receive palliative care exhibited higher comorbidity burden as measured by the CCI. This finding may reflect the complexity of disease trajectories in multimorbid patients, with potential negative consequences regarding the recognition of palliative care needs. In addition, comorbidities may also lead to more fragmented care increasing the likelihood of missed palliative care delivery. The observed pattern underscores the need for more systematic identification of palliative care needs, particularly in multimorbid patients with rapidly progressive diseases like ALS.

Clustering the care pathways of pALS with palliative care revealed substantial variability in care delivery regarding the type, order, and timing of services provided. This approach offered valuable insights into palliative care utilization. Notably, end-of-life care quality varied across clusters. For example a combination of outpatient palliative services, as observed in clusters 3 and 4, corresponded with higher end-of-life care quality compared to PPC alone (cluster 1). Additionally, palliative care pathways further revealed that initiating PPC before SPHC (cluster 3) corresponded with better outcomes than the reverse order (cluster 4). Similarly, while all pALS of clusters 6 and 7 had been provided with inpatient palliative care shortly before death, only the inclusion of outpatient services in cluster 7 went along with increased quality of end-of-life care. Importantly, according to our indicators quality of end-of-life care was highest in pathways with hospice care as their final type of palliative care service, often preceded by outpatient palliative care (cluster 5, 8, 9). One possible explanation is that engagement with outpatient services may facilitate early discussions about treatment decisions and advance care planning, ensuring that patients' preferences have been considered and documented. This may reduce unplanned hospital admissions and burdensome interventions at the end of life.

Compared to prior studies on ALS, which predominantly focused on analysis of primary data or qualitative surveys, this study adds a novel methodological perspective. Our approach allowed a nuanced understanding of the temporal dynamics in service types, which are often oversimplified in traditional analyses [15].

While this study provided valuable insights, it also raised important questions for further investigation. The causal factors behind palliative care pathways depicted in our study remain unknown. Future research should therefore investigate how the availability of palliative care services or individual preferences of pALS, informal caregivers and health care professionals shape palliative care delivery. The association of clinical characteristics such as disease duration, severity or ALS phenotypes with palliative care pathways and quality of end-of-life care also demand further investigation. As we have not explored the interactions of pALS with specialized neurologists here, additional research is needed to explore the role of interdisciplinary collaboration in palliative care pathways.

This study has several strengths that contribute to the growing body of knowledge on palliative care utilization in ALS and its association with end-of-life outcomes. By utilizing a population-based dataset of routinely collected administrative health claims, we were able to investigate patterns and outcomes in a large cohort compared to other national and international studies often relying on survey data of registries prone to strong selection and recall bias or response shift [41–45]. Through sequence analysis, we uncovered distinct clusters and provided nuanced descriptions of palliative care pathways, expanding current knowledge on palliative care delivery in pALS. Our results also highlight critical gaps in access to palliative care, with nearly half of the cohort not receiving any form of palliative care services.

Despite these strengths, our study has limitations. Administrative claims data, while robust for capturing healthcare utilization, do not include detailed clinical information such as functional status, symptom burden, or patient-reported outcomes. Indicators for measuring quality of end-of-life care were restricted to the last 30 days of life and could profit from enlargement and revision. Further, we acknowledge that these indicators reflect only a subset of quality dimensions and are inherently limited by the lack of contextual clinical and personal information in administrative datasets. We did not consider specialists, i.e. neurologists, not billing palliative care services. Moreover, our dataset lacked information on personal values and preferences potentially associated with choice of palliative care services. Access to data from more than one year before death, would have allowed us to describe the full care pathway from time of diagnosis onward and could possibly have revealed the initiation of palliative care services earlier, inducing pathways to be clustered differently. In addition, the study population was limited to individuals insured by BARMER, a major statutory health insurance provider. Although BARMER covers a broad and demographically diverse population, systematic differences in sociodemographic composition, regional distribution, or insurance-specific care incentives may limit generalizability to other populations [46]. Finally, while sequence analysis and clustering provided insights into palliative care pathways, the applied methods are computationally complex and may not be easily replicable in other datasets.

## Conclusion

This study highlighted the variability in palliative care delivery among pALS, with substantial differences in the type, order and timing of services provided. By applying sequence analysis, we introduced a novel method to examine palliative care pathways, uncovering nuanced patterns of service utilization. Our findings suggest that the quality of end-of-life care is not solely determined by the provision of palliative care and types of palliative care services but varies based on the sequence of services delivered. Future research should investigate the role of interdisciplinary collaboration in palliative care pathways and explore preferences and clinical characteristics of pALS to better understand causal factors influencing the quality of end-of-life care.

## Data Availability

Data underlying this study will be made available in anonymized form upon reasonable request from qualified investigators. Requests can be submitted to the corresponding author with information regarding the planned investigation and expected scientific value when adding data of this study, the institution in where the data will be processed.

## Abbreviations

ALS: Amyotrophic lateral sclerosis
pALS: People with amyotrophic lateral sclerosis
PPC: Primary palliative care
SPHC: Specialized palliative home care
CCI: Charlson Comorbidity Index
ICU: Intensive care unit
NW: Needleman-Wunsch
TNW: Temporal Needleman-Wunsch
